# Correlations among Corneal Biomechanical Parameters, Stiffness, and Thickness Measured Using Corvis ST and Pentacam in Patients with Ocular Hypertension

**DOI:** 10.1155/2022/7387581

**Published:** 2022-12-03

**Authors:** Qian Liu, Chenjiu Pang, Changgeng Liu, Wenjun Cheng, Shuai Ming, Wenwen Du, Xiaomei Feng

**Affiliations:** Henan Provincial People's Hospital, Henan Eye Hospital, Henan Eye Institute and Zhengzhou University People's Hospital, Zhengzhou, China

## Abstract

**Background:**

To preliminary explore the correlations among corneal biomechanical parameters, stiffness, and thickness in patients with ocular hypertension (OHT) before and after treatment with topical antiglaucoma medications.

**Methods:**

This was a retrospective study that included 35 eyes with newly diagnosed OHT. Axial length (AL), apical corneal thickness, and minimum corneal thickness were measured using Pentacam. The lengths, velocities, and times of the first and second corneal applanations (A1L, A1V, A1T, A2L, A2V, and A2T, respectively); the highest concavity radius; highest concavity peak distance (PDHC); highest concavity deformation amplitude (DAHC); highest concavity time (HCT); pachymetry (PACH); stress-strain index (SSI); stiffness parameter-A1 (SP-A1); deformation amplitude ratio (DA ratio); integrated radius (IR); Ambrosio's relational thickness horizontal (ARTh); corneal biomechanical index; noncorrected intraocular pressure (IOPnct); and biomechanically corrected IOP (bIOP) values were measured using the corneal visualization Scheimpflug technology (Corvis ST/CST).

**Results:**

After 5 weeks of treatment, Goldman applanation tonometer-IOP, IOPnct, bIOP, PACH, A1T, A2V, SSI, SP-A1, and ARTh decreased, but A1V, A2T, PDHC, DAHC, DA ratio, and IR increased significantly (all *p* < 0.05). SP-A1 and A1T were positively associated with premedication IOP and IOP changes, whereas A1V, A2T, PDHC, and IR were negatively associated (all *p* < 0.05). DAHC and DA ratio had significantly negative correlations with IOP variations. PDHC was found to be positively correlated with AL (*p* < 0.05). A positive relationship was noted between SP-A1 and HCT before medication (*p* < 0.05).

**Conclusions:**

SP-A1 was significantly and consistently associated with IOP. HCT might be correlated with SP-A1. SP-A1 and CST parameters could serve as potential biomarkers for evaluating OHT treatment efficacy.

## 1. Background

Glaucoma is a leading cause of blindness worldwide. Intraocular pressure (IOP) is the most important risk factor associated with the occurrence and progression of glaucoma [1]. Recently, attention toward corneal biomechanics has increased with regard to the diagnosis of glaucoma and post-treatment follow-up strategy. Alterations in the biomechanical properties of the cornea determine its deformation response to applanation, which may affect IOP and lead to the progression of glaucoma [2, 3]. Several studies have suggested different corneal biomechanical properties as important risk factors in glaucoma. Furthermore, IOP corrected according to corneal biomechanical properties improves measurement accuracy. The corneal visualization Scheimpflug technology (Corvis ST, CST) has been extensively employed to understand the corneal biomechanics of eyes with corneal diseases, glaucoma, or normal eyes [4, 5].

The CST is a noncontact device that can help investigate the corneal biomechanical properties in vivo [6]. The advantages of CST are excellent noncontact measurement of IOP, central corneal thickness (CCT), and corneal biomechanical parameters simultaneously [7]. A previous study found that the long-term use of some antiglaucoma medications might have an effect on corneal biomechanical properties; these medications might exert an indirect effect on these properties by reducing the magnitude of IOP [8]. However, most studies investigate corneal biomechanics in glaucoma based on corneal hysteresis, which is not associated with corneal stiffness.

Stiffness parameter-A1 (SP-A1) was introduced as a novel parameter for measuring corneal stiffness and rigidity using CST [9–11]. SP-A1 is a useful indicator of corneal resistance to deformation [12]. A higher corneal stiffness is considered to reflect a higher peripapillary scleral stiffness and thus a greater optic nerve head vulnerability [13]. Studies indicate that stiffness is a deformation parameter that could be derived by mapping a high-speed photograph series of corneal deformation responses to a calibrated air puff [10]. Initial studies have reported significant differences in SP-A1 between keratoconic and normal eyes. Most previous studies on glaucoma have explored corneal hysteresis as a corneal biomechanical property. In addition, a higher corneal stiffness parameter might be predictive of glaucomatous progression in glaucoma suspect eyes [13]. These results suggested that stiffness parameter variations might be potential influencing factors during glaucoma treatment and progression. However, few studies have been devoted to observe the dynamic corneal stiffness changes and to investigate whether corneal stiffness is altered with the treatment of ocular hypertension. Additionally, the correlations between corneal stiffness and other corneal properties in ocular hypertension (OHT) eyes remain unclear.

In the present study, we explored the changes in and relationships among corneal biomechanical parameters, stiffness, and thickness after antiglaucoma pharmacotherapy in patients with OHT. Furthermore, we aimed to assess and compare the stiffness parameters with the associated factors and to evaluate the feasibility of using SP-A1 for monitoring glaucoma treatment and progression.

## 2. Methods

In this retrospective study, we reviewed the medical records of consecutive patients who underwent antiglaucoma pharmacotherapy in the glaucoma division of Henan Eye Hospital and Henan Eye Institute from December 2020 to November 2021. This study was conducted in compliance with the Declaration of Helsinki for research involving human participants. Institutional Review Board approval was obtained from Henan Eye Hospital [HNEECKY-2021(47)]. Informed consent was obtained from all patients.

### 2.1. Inclusion and Exclusion Criteria

This study included 35 eyes from 22 consecutive patients who were aged >18 years and newly diagnosed with OHT. OHT was defined as having a normal visual field (VF) with an untreated Goldman applanation tonometer-IOP (GAT-IOP) of >21 mmHg that had not previously been treated with topical or systemic medications, laser technology, or surgery [14]. Patients were excluded if they had any other ocular or neurological comorbidities or if they had previously undergone ocular treatment that could affect corneal biomechanics. Patients with secondary glaucoma caused by factors such as uveitis, trauma, steroids, or corneal scarring were excluded. Patients with hypermetropia or myopia >5.0 diopters (D) and/or astigmatism >3.0 D were excluded.

### 2.2. Clinical Examination

The measurements were performed between 9:00 AM and 4:00 PM by an experienced operator. The measurement of best corrected visual acuity (BCVA), slit lamp examination, and fundus examination were performed by a professional glaucoma specialist. IOP was tested using a GAT before and after treatment with topical medications for 5 weeks, and the axial length (AL) in all participants was measured using partial coherence interferometry (IOLMaster, Zeiss, Germany) [15].

All patients' eyes were examined using a Pentacam imaging system (70900, OCULUS, Germany) while they fixated their eyes on a target light source. To ensure the image quality, only results that were marked as “OK” on the display specification window were adopted. Pachymetry (PACH) assessments and measurement of corneal deformation parameters were performed using CST (OCULUS 72100, Wetzlar, Germany). CST records the entire dynamic reaction of the cornea to a fixed air impulse. To ensure correct alignment, the patients were asked to place their heads on the chin rest such that the puff nozzle was centered on the eye.

### 2.3. Data Collection

From the medical records of all patients, data on demographic characteristics, including age, sex, systemic health condition, and lens status, were collected. Clinical data on IOP, VF (Humphery, Carl Zeiss, Germany), BCVA, and corneal thickness were collected before and after treatment. CCT, apical corneal thickness (ACT), and minimum corneal thickness (MCT) were measured using Pentacam [16]. Using CST, the lengths, velocities, and times of the first and second corneal applanations (A1L, A1V, A1T, A2L, A2V, and A2T, respectively), as well as the highest concavity radius (HCR), highest concavity peak distance (PDHC), highest concavity deformation amplitude (DAHC), and highest concavity time (HCT) were observed. The stress-strain index (SSI) and SP-A1 were also measured. New CST parameters were considered, such as the deformation amplitude ratio (DA ratio), integrated radius (IR), and Ambrosio's relational thickness horizontal (ARTh). IR was the integrated area under the radius of the inversed curvature during the concave phase. ARTh was defined as the ratio between the thickest and thinnest point. It is a progression index that describes the increase in thickness from the thinnest point to the periphery [11]. Noncorrected IOP (IOPnct) and biomechanically corrected IOP (bIOP) were recorded using CST and considered as CST-IOP parameters.

### 2.4. Statistical Analysis

Statistical analysis was performed using IBM SPSS statistics (version 19.0.0). The Kolmogorov–Smirnov normality test was used. Clinical characteristics are presented as mean ± standard deviation (SD). Paired *t*-test was used to investigate the differences in clinical parameters and changes of the first and second corneal applanations between two time points: baseline and post-treatment. Multiple linear regression analyses were performed to determine associations between the parameters. In all analyses, *p* < 0.05 was considered statistically significant. For the clinical parameter data with *p* < 0.05, the statistical power analysis was performed using *G*^*∗*^ Power (version 3.1.9.7), and all parameters power >0.96, except SSI and ARTH, which were 0.757 and 0.527, respectively. For GAT-IOP, IOPnct, and bIOP, *p* < 0.0167 was considered significant in the paired *t*-test.

## 3. Results

A total of 35 eyes of patients with newly diagnosed OHT were included in this study. The mean age of the participants was 28.86 ± 10.22 years, and 36.37% of the participants were women. Furthermore, 13 of the 35 patients had binocular parameters. The mean number of anti-glaucoma medications used for treatment was 1.06 ± 0.24. None of the included patients had a history of glaucoma surgery or antiglaucoma pharmacotherapy (Table 1).

The GAT-IOP decreased significantly from 25.69 ± 4.63 mmHg at baseline to 17.03 ± 3.74 mmHg after treatment (*p* < 0.001, Table 2). GAT-IOP and IOPnct were significantly higher than the corrected bIOP at baseline (*p*=0.03 and *p* < 0.001, respectively; not shown in Table 2). However, there were no significant differences in GAT-IOP and CST-IOP parameters after treatment. There was no significant change in BCVA and AL after treatment (*p* > 0.05, Table 2). CCT, ACT, and MCT mildly decreased (all *p* > 0.05) after treatment in all patients. We found that PACH serves as a proxy for CST-CCT and that it decreased significantly after treatment (*p*=0.014, Table 2).

### 3.1. Corneal Biomechanical Parameters and Stiffness Measured Using CST

Changes in CST parameters in patients with OHT after treatment are shown in Table 3. The mean SP-A1 was 109.08 mmHg/mm (SD, 11.79 mmHg/mm) after treatment, which was significantly lower than the baseline level (*p* < 0.001, Table 3). Similarly, SSI decreased significantly after treatment (*p* = 0.010, Table 3). Moreover, A1V, A2T, PDHC, DAHC, DA ratio, and IR increased significantly after treatment (all *p* < 0.05). On the contrary, A1T, A2V, PACH, and ARTh were significantly lower at follow-up than at baseline (all *p* < 0.05, Tables 2 and 3). However, there were no significant changes in A1L, A2L, HCR, and HCT (all *p* ≥ 0.05). The changes of first and second corneal applanations (AV and AT) in the baseline compared to the changes of first and second corneal applanations (AV and AT) in post-treatment varied significantly (*p* < 0.001, Table 3).

### 3.2. Association between Corneal Biomechanical Parameters and Clinical Characteristics

Independent factor analysis showed that SP-A1 and A1T were positively correlated with pre-IOP and changes in IOP; however, A1V, A2T, PDHC, and IR were negatively correlated with pre-IOP and changes in IOP after anti-glaucoma treatment (*p* < 0.05). Changes in AV were positively correlated with pre-IOP and negatively correlated with changes in IOP (*p*<0.05). DAHC showed significant negative correlations with changes in IOP (*p* < 0.01, Table 4). The longer the AL, the higher the PDHC (*p* = 0.015, Table 4). SP-A1 was positive with Pentacam-derived CCT, whereas A1V and IR were negative (*p* < 0.001, Table 4).

### 3.3. Association between Corneal Stiffness Parameters and the Highest Concavity Parameters

The highest concavity (HC) referred to the HC of the cornea in response to the air puff. The relationships between variations in stiffness parameters after treatment and HC CST-acquired values were analysed. A statistically significant linear relationship was noted between SP-A1 and corneal biomechanical parameters (*p* < 0.05). A positive linear relationship was noted between SP-A1 and HCT at baseline (*p*=0.032, Table 5). Additionally, there was no significant correlation between SSI and HC parameters (*p* > 0.05, Table 5).

## 4. Discussion

A previous study reported that peripapillary stiffness was higher in glaucomatous eyes than in normal eyes [17]. Moreover, eyes with normal-tension glaucoma were reported to show a lower SP-A1 than normal eyes; however, these results were confounded by a lower IOP and the use of topical medications [14]. In the current study, SP-A1 and SSI indicated that changes in corneal tissue stiffness decreased significantly after treatment in OHT eyes. Moreover, a decrease in SP-A1 indicated that the cornea was more deformable after antiglaucoma pharmacotherapy.

A higher corneal stiffness is considered to reflect a higher peripapillary scleral stiffness and thus a greater optic nerve head vulnerability [13]. In this study, multiple linear regression analysis showed that a higher IOP might be associated with a higher SP-A1 after treatment. The remarkable decrease in IOP was associated with a decrease in SP-A1. The present results suggested that SP-A1 might be very sensitive to IOP changes and that it is significantly and consistently associated with IOP. SP-A1 could probably provide more information about glaucoma treatment and follow-up strategy. In a previous study, a higher SP-A1 was found in eyes with a higher IOP and thicker CCT [18]. Similarly, in this study, there was a significant association between SP-A1 post-treatment and Pentacam-derived CCT. These results suggested that patients with thicker CCT might more easily achieve a higher SP-A1 post-treatment, which is a useful indicator of corneal resistance to deformation. HC described the deformability of cornea. In the current study, the lower HCT before treatment was correlated with decreasing SP-A1. The outcomes of the present study showed that SP-A1 reflects corneal stiffness and is closely related to corneal properties. The two parameters could share corroboration in evaluating treatment efficacy and predicting progression of glaucomatous optic nerve injuries.

In the present study, in addition to other CST parameters, A1V, A2T, IR, and PDHC showed a significant increase after treatment. Similar findings were noted as effects of antiglaucoma medications in a previous study [8]. Serbecic et al. found A1, HC, and A2 time points showed excellent repeatability and reproducibility [19]. A previous study reported that A1V was significantly higher in eyes treated with prostaglandin analogues (PGAs) than in those treated without PGAs [2]. Another study reported that A1V increased significantly even after the first week of antiglaucoma surgery [20]. In some studies on long-term treatment with topical medications, DAHC increased markedly after 2 years, while PDHC increased slightly [2, 8]. In other studies, PDHC and CRF increased significantly after trabeculectomy or other antiglaucoma surgeries [21]. Partially consistent with these studies, the present study noted a significant increase in PDHC, DAHC, and DA ratio. Similar to previous studies [8], the present study noted that post-treatment A1V (m/s) was higher in patients with newly diagnosed OHT. Moreover, A2V decreased significantly whereas A2T increased after pharmacotherapy and surgery in previous studies [8, 21]. Additionally, changes in A2V were reported to gradually slow down over time [21]. In this study, SSI and SP-A1 showed corneal stiffness changes and decreased significantly after pharmacotherapy. Consistent with the findings of previous research, A1L, A2L, HCT, and HCR infrequently showed no significant changes after long-term pharmacotherapy or at an early stage after surgery [2, 8, 21, 22].

Asaoka et al. found that A1T, A1V, A2V, PDHC, and DAHC were influenced by IOP [23]. Huseynova et al. reported that IOPcc was positively correlated with A1T and A2V and negatively correlated with A2T and A1V. In the present study, the pretreatment IOP was positively associated with SP-A1 and negatively associated with A1V. A decrease in IOP was significantly associated with variations in corneal parameters such as A1V, PDHC, and SP-A1. Moreover, IOP showed a positive correlation with SP-A1 and a negative correlation with A1V, IR, and PDHC. These results suggested that a variation in IOP may lead to changes in corneal stiffness and biomechanical properties in the early stage of antiglaucoma pharmacotherapy. The velocity parameters reflected corneal elasticity and were associated with the cross-linking of collagen fibrils in the cornea [24]. A decrease in IOP might enhance the elasticity and viscosity of the cornea and induce a strong pressure threshold [25]. A lower post-treatment IOP led to a smaller IR and PDHC magnitude in this study. A possible explanation for this result is that perhaps glaucoma medications, such as PGAs, activated matrix metalloproteinases (MMPs) and suppressed their tissue inhibitors to increase the keratocyte density in the corneal stroma. This PGA-induced corneal tissue remodeling may influence DAHC and PDHC [26]. The changes in DAHC at an early stage after pharmacotherapy indicate that a variation in corneal properties may occur earlier than expected. IR and ARTh served as new CST parameters that were rarely discussed during glaucoma treatment. In this study, IR increased significantly postmedication, while the higher IOP before medication induced the lower IR after treatment. ARTh was found to be significantly decreased in the present study, and a lower value indicated a thinner cornea and/or a faster thickness increase toward the periphery [11]. These results suggested that an IOP decrease under effective treatment may result in changes in the characteristic parameters of the cornea.

Previous studies have shown that clinical and demographic factors affect corneal dynamics. Huang et al. found that a decrease in AL following a reduction in IOP might be involved in the dynamic reaction of the cornea [21]. These results showed that AL is related to tissue extension, ocular rigidity [27], and biomechanical properties. In contrast to prominent AL changes after surgical treatment in a previous study [8], there were no significant differences in AL after pharmacotherapy in the current study. However, in this study, a longer AL was associated with a larger PDHC after a decrease in IOP. AL suggested multiple potential factors that impact corneal biomechanics. In cornea, a stiffer appearance and higher CCT are accompanied by less deformation in response to air pulse [28]. A few studies have evaluated a decrease in the changes in CCT following long-term topical pharmacotherapy [29, 30]. Activation of MMPs induces the degradation of the corneal stromal extracellular matrix, which ultimately leads to a reduction in CCT [29, 30]. However, some studies observed no significant changes in CCT during a 2-year treatment with PGA [8]. In the present study, the CCTs measured using Pentacam were stable during the short-term follow-up period. However, PACH, as a proxy of CST-CCT, showed a significant decrease. The present results suggest that PACH, as a proxy of CST-CCT, might overestimate Pentacam CCT, especially when the IOP is high. Kumar et al. found the coefficient of variation (COV) of CCT measurements of Corvis were lower than those of Pentacam [31]. It was a complicated factor that could affect corneal thickness, and the exact mechanisms must be elucidated in the future.

The consistency between GAT-IOP and CST-IOP has been evaluated in patients with and without glaucoma in previous studies; some showed no significant differences [32, 33], while one study showed that CST-IOP was higher than GAT-IOP in patients who underwent LASIK [34]. In this study, GAT-IOP was consistent with IOPnct; however, the former decreased significantly after biomechanical correction before pharmacotherapy, and these results were similar to those of previous reports. No significant differences were found among GAT-IOP, IOPnct, and bIOP after topical antiglaucomatous therapy. These results suggest that IOP tested by CST might underestimate GAT-IOP, especially when the IOP is high [7].

This study has some limitations. First, the retrospective study design may have led to bias. Second, the number of patients was small. Further prospective studies with a larger number of patients and different types of glaucoma medications are needed.

## 5. Conclusions

In summary, significant decreases in corneal stiffness were noted in patients with OHT after antiglaucoma therapy. SP-A1 was significantly and consistently associated with IOP in OHT eyes. The corneal properties HCT and CCT were significantly associated with SP-A1. SP-A1 measured using CST could be a potential biomarker for evaluating the treatment efficacy and disease progression in OHT eyes. As new CST parameters, IR significantly increased and ARTh decreased post antiglaucoma therapy. IR was negatively associated with IOP and CCT. Future studies would contribute to a broader understanding of the correlations among corneal biomechanical properties, stiffness, and thickness after IOP-lowering therapy.

## Figures and Tables

**Table 1:** Baseline patient and ocular characteristics.

Characteristic	Summary statistics (*N* = 35 eyes in 22 patients)
Age (at time of surgery)	
Mean (standard deviation)	28.86 (10.22)
Gender	
Male	14 (63.63%)
Female	8 (36.37%)
Diabetics	
No	100 (100%)
Yes	0 (0%)
Hypertension	
No	100 (100%)
Yes	0 (0%)
Number of eyes enrolled in study	
1	9 (40.91%)
2	13 (59.09%)
Numbers of OHT eyes on glaucoma medication	1.06 ± 0.24
Tafluprost	29
CA-inh and *β*-blockers (brinzolamide and timolol)	4
*α* agonist	4
Prior glaucoma surgery	
No	100 (100%)
Yes	0 (0%)
Prior cataract surgery	
No	100 (100%)
Yes	0 (0%)
Intraocular pressure (Goldman applanation tonometry)	25.69 (4.63)
Intraocular pressure (Corvis ST, uncorrected)	23.53 (7.47)
Intraocular pressure (Corvis ST, corrected)	22.59 (6.61)

OHT: ocular hypertension; CA-inh: carbonic anhydrase inhibitors. Data are mean (standard deviation) unless otherwise indicated.

**Table 2:** Clinical characteristics in OHT patients with glaucoma medication treatment.

	Baseline (mean (SD), *n*)	Eyes with topical treatment (mean (SD), *n*)	*p* value^*∗*^
GAT IOP (mmHg)	25.69 (4.63), 35	17.03 (3.74), 35	**<0.001** ^**#**^
IOPnct (mmHg)	23.53 (7.47), 35	17.70 (2.84), 35	**<0.001** ^**#**^
bIOP (mmHg)	22.55 (6.62), 35	17.37 (2.44), 35	**<0.001** ^**#**^
BCVA (LogMAR)	0.24 (0.34), 35	0.23 (0.32), 35	0.97
Axial lengths (AL)	24.48 (1.38), 25	24.45 (1.37), 25	0.18
Corneal thickness in central pupil	545.71 (33.95), 35	544.31 (34.25), 35	0.096
Apex corneal thickness	545.74(33.52), 35	544.31 (32.42), 35	0.091
Minimum corneal thickness	541.54 (34.25), 35	540.23 (32.71), 35	0.108
PACH (*μ*m)	547.34 (34.23), 35	544.29 (34.63), 35	**0.014** ^**#**^

^*∗*^Paired *t*-test for comparison of any difference at baseline and after medication. OHT: ocular hypertension; SD: standard deviation; GAT-IOP: Goldman applanation tonometer intraocular pressure; IOPnct: noncorrected IOP; bIOP: biomechanical corrected IOP value; BCVA: best corrected visual acuity, PACH: pachymetry, stands for CST corneal central thickness (CST CCT). ^#^Significant differences were found by *p* < 0.05.

**Table 3:** Corvis ST parameters before and after glaucoma medication treatment.

Factors	Baseline (mean ± SD)	Eyes with topical treatment (mean ± SD)	*p* value^*∗*^
A1L (mm)	2.33 ± 0.26	2.34 ± 0.34	0.937
A1V (m/s)	0.11 ± 0.02	0.14 ± 0.02	**<0.001** ^**#**^
A1T (ms)	8.38 ± 1.03	7.55 ± 0.38	**<0.001** ^**#**^
A2L (mm)	2.10 ± 0.44	2.06 ± 0.37	0.565
A2V (m/s)	−0.21 ± 0.05	−0.26 ± 0.02	**<0.001** ^**#**^
A2T (ms)	21.32 ± 0.96	21.97 ± 0.48	**0.002** ^**#**^
Changes AL (A1L-A2L)	0.23 ± 0.44	0.25 ± 0.51	**0.32**
Changes AV (A1V-A2V)	0.32 ± 0.06	0.41 ± 0.04	**<0.001** ^**#**^
Changes AT (A1T-A2T)	−12.94 ± 1.81	−14.42 ± 0.67	**<0.001** ^**#**^
PDHC (mm)	4.36 ± 0.57	4.91 ± 0.24	**<0.001** ^**#**^
HCR (mm)	7.69 ± 1.12	7.48 ± 0.82	0.236
DAHC (mm)	0.88 ± 0.1	1.04 ± 0.08	**<0.001** ^**#**^
DA ratio	3.70 ± 0.64	4.07 ± 0.44	**0.002** ^**#**^
HCT (ms)	17.36 ± 0.46	17.29 ± 0.54	0.560
SSI	1.03 ± 0.22	0.94 ± 0.15	**0.010** ^**#**^
SP-A1	128.35 ± 17.85	109.08 ± 11.79	**<0.001** ^**#**^
IR	6.72 ± 1.54	7.91 ± 1.04	**<0.001** ^**#**^
ARTH	531.2 ± 98.29	499.30 ± 18.55	**0.004** ^**#**^

^*∗*^Paired *t*-test was used in comparison of any difference across time points adjusted. Data were showed in the form of Mean (standard deviation). A1L: applanation1 1ength; A1V: applanation1 velocity; A1T: applanation 1 time; A2L: applanation2 1ength; A2V: applanation2 velocity; A2T: applanation 2 time; Changes AL (A1L-A2L): A1L minus A2L; Changes AV (A1V-A2V): A1L minus A2L; Changes AT (A1T-A2T): A1L minus A2L; PDHC: highest concavity peak distance; HCR: highest concavity radius; DAHC: highest concavity def amp; HCT: highest concavity time; SSI: stress-strain index; IOP: intraocular pressure; SP-A1: stiffness parameter at first applanation; IR: inter radius; ARTH: amobrosio relational thickness horizontal. ^#^Significant differences were found by *p* < 0.05.

**Table 4:** Multiple line regression analysis associated factors for significant Corvis ST parameters.

Dependent variable	Independent variable	Standardized regression coefficient	*p* ^*∗*^
A1V	Pre-IOP	−2.85	**<0.001** ^*∗*^
	Changes in IOPs	−3.33	**<0.001** ^*∗*^
	Axial lengths	−0.04	0.771
	Corneal thickness in central pupil	−0.35	**0.021** ^*∗*^

A1T	Pre-IOP	2.03	**0.007** ^*∗*^
	Changes in IOPs	2.53	**0.002** ^*∗*^
	Axial lengths	−0.13	0.449
	Corneal thickness in central pupil	0.32	0.062

A2T	Pre-IOP	−1.99	**0.020** ^*∗*^
	Changes in IOPs	−2.25	**0.011** ^*∗*^
	Axial lengths	−0.28	0.152
	Corneal thickness in central pupil	0.16	0.400

Changes in AV	Pre-IOP	0.008	**0.044** ^*∗*^
	Changes in IOPs	−0.015	**0.000** ^*∗*^
	Axial lengths	0.011	0.072
	Corneal thickness in central pupil	−0.001	**0.003** ^*∗*^

Changes in AT	Pre-IOP	−0.017	0.690
	Changes in IOPs	0.285	**0.000** ^*∗*^
	Axial lengths	−0.103	0.113
	Corneal thickness in central pupil	0.002	0.608

PDHC	Pre-IOP	−1.83	**0.005** ^*∗*^
	Changes in IOPs	−2.22	**0.001** ^*∗*^
	Axial lengths	0.37	**0.015** ^*∗*^
	Corneal thickness in central pupil	0.05	0.736

DAHC	Pre-IOP	−1.53	0.050
	Changes in IOPs	−1.98	**0.015** ^*∗*^
	Axial lengths	0.23	0.216
	Corneal thickness in central pupil	−0.17	0.343

DA ratio	Pre-IOP	−1.19	0.070
	Changes in IOPs	−1.51	**0.026** ^*∗*^
	Axial lengths	−0.16	0.309
	Corneal thickness in central pupil	−0.80	**<0.001** ^*∗*^

SP-A1	Pre-IOP	1.39	**0.030** ^*∗*^
	Changes in IOPs	1.88	**0.005** ^*∗*^
	Axial lengths	0.07	0.648
	Corneal thickness in central pupil	0.74	**<0.001** ^*∗*^

Inter radius	Pre-IOP	−1.48	**0.023** ^*∗*^
	Changes in IOPs	−1.90	**0.005** ^*∗*^
	Axial lengths	0.02	0.904
	Corneal thickness in central pupil	−0.77	**<0.001** ^*∗*^

All the independent variable from liner regression analysis were included in the model. Pre-IOP: Pre-Goldman intraocular pressure before antiglaucomatous medications application. Changes in IOPs: changes in Goldman intraocular pressure after antiglaucomatous medications applications. A1V: applanation1 velocity; A1T: applanation1 time; A2T: applanation2 time; Changes in AV: changes in A1V-A2V after anti glaucomatous medications applications; Changes in AT: changes in A1T-A2T after antiglaucomatous medications applications; PDHC: highest concavity peak distance; DAHC: highest concavity deformation amplitude; SP-A1: stiffness parameter A1. ^*∗*^Multiple liner regression analysis of different independent variable with *p* < 0.05.

**Table 5:** Multiple line regression analysis of corneal stiffness and highest concavity parameter.

Dependent variable	Independent variable	Standardized regression coefficient	*p* ^*∗*^
SP-A1	Pre-PDHC	−0.38	0.460
	Pre-HCR	−0.06	0.709
	Pre-DAHC	0.58	0.264
	Pre-HCT	0.37	**0.032** ^*∗*^

SSI	Pre-PDHC	−0.21	0.700
	Pre-HCR	0.22	0.221
	Pre-DAHC	0.33	0.550
	Pre-HCT	0.29	0.107

All the independent variable from liner regression analysis were included in the model. PDHC: highest concavity peak distance; DAHC: highest concavity def amp. HCR: HCR: highest concavity radius; HCT: highest concavity time; SP-A1: stiffness parameter A1, SSI: stress-strain index. ^*∗*^Liner regression analysis of different independent variable with *p* < 0.05.

## Data Availability

The datasets used and/or analysed during the current study are available from the corresponding author upon reasonable request.

## Abbreviations

OHT: Ocular hypertension
AL: Axial length
ACT: Apical corneal thickness
MCT: Minimum corneal thickness
A1L, A1V, A1T, A2L, A2V, and A2T: The lengths, velocities, and times of first and second corneal applanations
HCR: Highest concavity radius
PDHC: Highest concavity peak distance
DAHC: Highest concavity deformation amplitude
HCT: Highest concavity time
PACH: Pachymetry
SSI: Stress-strain index
SP-A1: Stiffness parameter-A1
DA ratio: Deformation amplitude ratio
IR: Integrated radius
ARTh: Ambrosio's relational thickness horizontal
IOPnct: Non-corrected intraocular pressure
bIOP: Biomechanically corrected IOP
Corvis ST/CST: Corneal visualization Scheimpflug technology
GAT-IOP: Goldman applanation tonometer-intraocular pressure
IOP: Intraocular pressure
CCT: Central corneal thickness
VF: Visual field
D: Diopter
BCVA: Best corrected visual acuity
SD: Standard deviation
HC: Highest concavity
PGA: Prostaglandin analogue
MMP: Matrix metalloproteinase.
